# Cotton pest management practices and the selection of pyrethroid resistance in *Anopheles gambiae *population in Northern Benin

**DOI:** 10.1186/1756-3305-4-60

**Published:** 2011-04-13

**Authors:** Anges Yadouleton, Thibaud Martin, Gil Padonou, Fabrice Chandre, Alex Asidi, Luc Djogbenou, Roch Dabiré, Rock Aïkpon, Michel Boko, Isabelle Glitho, Martin Akogbeto

**Affiliations:** 1 Centre de Recherche Entomologique de Cotonou (CREC), 06 BP 2604 Cotonou, République du Bénin; 2 Centre de Coopération Internationale en Recherche Agronomique pour le Développement, CIRAD-UR Hortsys, 34980 Montpellier, France; 3 Institut de Recherche pour le Développement (IRD)/Laboratoire de lutte contre les Insectes Nuisibles (LIN), Centre Collaborateur OMS 911 Ave Agropolis BP 64501, 34394 Montpellier Cedex 5 France; 4 Institut de Recherche pour le Développement (IRD)/Centre de Recherche Entomologique de Cotonou (CREC), 01 BP 4414 RP Cotonou, République du Bénin; 5 Institut de Recherche en Science de la Santé (IRSS)/Centre Muraz, BP 390, Bobo-Dioulasso, Burkina Faso; 6 Université d'Abomey-calavi, Department of geography, B.P. 526 Abomey-Calavi, 01 BP 526 Cotonou, Benin; 7 Université de Lomé, Faculté de Sciences et Techniques, Lomé, Togo

## Abstract

**Background:**

Pyrethroid insecticides, carbamate and organophosphate are the classes of insecticides commonly used in agriculture for crop protection in Benin. Pyrethroids remain the only class of insecticides recommended by the WHO for impregnation of bed nets. Unfortunately, the high level of pyrethroid resistance in *Anopheles gambiae *s.l., threatens to undermine the success of pyrethroid treated nets. This study focuses on the investigation of agricultural practices in cotton growing areas, and their direct impact on larval populations of *An. gambiae *in surrounding breeding sites.

**Methods:**

The protocol was based on the collection of agro-sociological data where farmers were subjected to semi-structured questionnaires based on the strategies used for crop protection. This was complemented by bioassay tests to assess the susceptibility of malaria vectors to various insecticides. Molecular analysis was performed to characterize the resistance genes and the molecular forms of *An. gambiae*. Insecticide residues in soil samples from breeding sites were investigated to determine major factors that can inhibit the normal growth of mosquito larvae by exposing susceptible and resistant laboratory strains.

**Results:**

There is a common use by local farmers of mineral fertilizer NPK at 200 kg/ha and urea at 50 kg/hectare following insecticide treatments in both the Calendar Control Program (CCP) and the Targeted Intermittent Control Program (TICP). By contrast, no chemicals are involved in Biological Program (BP) where farmers use organic and natural fertilizers which include animal excreta.

Susceptibility test results confirmed a high resistance to DDT. Mean mortality of *An. gambiae *collected from the farms practicing CCP, TICP and BP methods were 33%, 42% and 65% respectively. *An. gambiae *populations from areas using the CCP and TICP programs showed resistance to permethrin with mortality of 50% and 58% respectively. By contrast, bioassay test results of *An. gambiae *from BP areas gave a high level of susceptibility to permethrin with an average mortality of 94%.

Molecular analysis identified *An. gambiae *s.s, and *An. arabiensis *with a high predominance of *An. gambiae s.s *(90%). The two molecular forms, M and S, were also determined with a high frequency of the S form (96%).

The *Kdr *gene seemed the main target- site resistance mechanism detected in CCP, TICP, and BP areas at the rates ranging from 32 to 78%. The frequency of *ace-1R *gene was very low (< 0.1).

The presence of inhibiting factors in soil samples under insecticide treatments were found and affected negatively in delaying the development of *An. gambiae *larval populations.

**Conclusions:**

This research shows that *Kdr *has spread widely in *An. gambiae*, mainly in CCP and TICP areas where pyrethroids are extensively used. To reduce the negative impact of pesticides use in cotton crop protection, the application of BP-like programs, which do not appear to select for vector resistance would be useful. These results could serve as scientific evidence of the spread of resistance due to a massive agricultural use of insecticides and contribute to the management of pesticides usage on cotton crops hence reducing the selection pressure of insecticides on *An. gambiae *populations.

## Background

Malaria remains a major public health problem in Africa. It is reported to be the most significant cause of morbidity and mortality, resulting in a critical loss of working days [1].

More than 2 billion people in the world are at risk of contracting malaria and one million deaths are recorded yearly of which 90% occur in Sub-Saharan Africa [1]. In Benin, malaria is still the most important disease leading to 67% consultations in local health centres [2]. The strategy of the National Malaria Control Programme (NMCP) is based on effective case management and vector control with Insecticide-Treated Nets (ITN) and Indoor Residual Spraying (IRS).The development of new insecticides for public health use is limited and requires enormous capital and time, making industry reluctant to embark on such ventures. Novel compounds or alternatives are to be sought in the agricultural pesticide pipeline. Several reports have recently shown evidence that the main African malaria vector, *Anopheles gambiae *s.l., has developed a high level of resistance to pyrethroid insecticides as well as to other classes of public health insecticides. While resistance is now spreading throughout Sub-Saharan Africa, reports from Benin and the West African region indicated the highest recorded frequencies of the resistance genes [3-6].

The development of pyrethroid resistance in the primary malaria vectors, *An. gambiae *s.l. and *An. funestus *[7] is a serious concern. In the last decade, the emergence of resistance in populations of *An. gambiae *to common classes of insecticides used in public health has been reported in many African countries including Kenya [8], Côte d'Ivoire [9], Benin [10-14], Niger [15], Burkina Faso [16,17], Mali[18], Nigeria [19], South Africa [20], and Cameroun [21]. In the 1960s, the role of selective treatment with organochlorines (OC) in agriculture on resistance of *An. gambiae *was observed in Mali [22]. Evidence of an association between agricultural use of insecticides and the emergence of resistance in malaria vectors has been repeatedly reported. In Côte d'Ivoire and Burkina Faso, N'Guessan *et al. *[23] reported that the level of vector resistance to pyrethroid insecticides increased during the cotton growing season. Higher frequencies of *kdr *alleles were observed in the more intensely farmed cotton production areas of Côte d'Ivoire [9]. In Bukina-Faso, a survey of *kdr *alleles in *An. gambiae *field populations showed also a higher frequency of *kdr *alleles in older cotton areas with a decreasing gradient to non treated areas [17].

Cotton crop protection represents 90% of the insecticide use in West Africa. The control strategies implemented against cotton pest especially *Helicoverpa armigera *required a regular repeated applications of insecticides during the cotton plant growing cycle. As recommended by the Institut National des Recherches Agronomiques du Benin (INRAB), six consecutive treatments are applied at two weeks interval to protect the crop against bollworms, leafworms and sucking pests. These insecticides are essentially composed of pyrethroids (PYs), organophosphates (OPs) which are also the main classes used in public health and a cyclodiene. The majority of cotton farms observed in northern Benin are located in the upland landscape while the lowland covers the major mosquito breeding sites. Thus run-off has been assumed to be the mechanism by which insecticides from agricultural sites reach malaria vector breeding sites, where they exert a huge selection pressure on larval stages of *An. gambiae *s.l. The main malaria vector *An. gambiae *breeds in puddles, stagnant pools and various sites around or within the lowland. During the rainy season, insecticide residues are washed downwards into mosquito breeding sites thus affecting larval population [24]. According to Akogbeto *et al. *[25] in Benin, insecticide treatments against cotton pests are applied twice a month, for a timeframe of three consecutive months (between July and October) each year. These treatment periods coincide with the rainy season and correspond to the period of high mosquito densities. The evidence supports the hypothesis that breeding sites contamination is the result of the coincidence of agricultural pesticide application and seasonal rainfall/runoff.

Alternatively, integrated pest and vector management (IPVM) strategies based on the rational use of chemical protection, has undoubtedly reduced the negative impact of pesticides on humans, and their environment, including the breeding sites of malaria vectors.

This study aimed to assess the impact of control strategies used against cotton pests (relative amount of insecticide) on the frequency and spread of insecticide resistance in *An. gambiae *populations. The study, conducted in northern Benin, compared the BP cotton cultivation sites (absence of pesticides use) with the CCP and TICP cotton cultivation sites where insecticides are extensively used. The study focused on the investigation of agricultural practices using pesticides for the control of cotton pests and their impact on the insecticide susceptibility of *An. gambiae *populations from surrounding breeding sites.

## Methods

### Cotton pests control strategies

Three pest management and control strategies are officially recommended in Benin:

(1) The Calendar Control Program (CCP) is based on the conventional treatment which systematically uses the full dosage of insecticides.

(2) The Targeted Intermittent Control Program (TICP) is based on two steps of crop protection [26,27]. The first steps is a protection which follows a conventional pesticide application schedule (every 14 days from the appearance of floral organs), but only the half dose of insecticide are usually applied. The second step includes a modification of the first treatment meaning that the half-dose left over during the earlier observation made the day before treatment would suggest that the pest populations exceed the economic thresholds of damage. The program was established five years ago.

(3) In the biological control program (BP), no chemical is used for plant protection. That program started over the past five years.

The area of the farms applying CCP and TICP was about 4 hectares and usually farmers ploughed sometime individually or work in groups of farmers' organizations. However, in BP sites of 1 hectare, farmers worked under the supervision of technicians from the Beninese Organization for Organic Farming Promotion (OBEPAP) who assisted in the implementation and the survey of good agricultural practices on organic cotton. These areas were characterised by a continual production of cotton crop.

### Study sites

**Figure 1:**
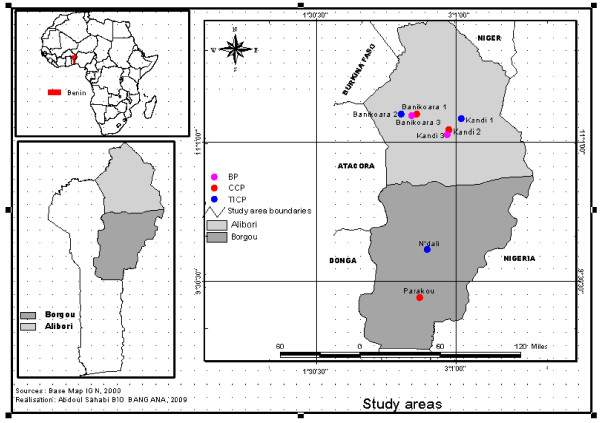
**Map of Benin showing the study sites**.

The study was conducted in the cotton areas around 8 cities in Benin (Figure 1). The choice of these areas took into account the various strategies of pest control. Semi permanent breeding sites were found in cotton fields where farmers used:

• the CCP around Parakou (2°62 E, 9°33 N), Kandi (2°95 E, 11°16 N) and Banikoara (2°59 E, 11°31 N). This pest management program started thirty years ago and was the main strategy against pest control used by more than 95% of the cotton farmers.

• the TICP around N'dali (2°70 E, 9°84 N), Kandi (3°08 E, 11°27 N) and Banikoara (2°41 E, 11°31 N) started five years ago and applied by 4% of cotton farmers.

• the BP around Kandi (2°92 E, 11°09 N) and Banikoara (2°52 E, 11°29 N) started five years ago and practiced by 1% of cotton farmers.

The annual mean rainfall recorded was about 1,300 mm yearly and characterized by a Sudanian climate with one rainy season (middle of May to October) and one dry season (November-May).

### KAP Study on the use of insecticides in cotton farms

To generate adequate information on the use of insecticide on cotton fields, Knowledge Attitude-Practice (KAP) surveys were organized in the study sites. In each site, leaders of farmer's organizations were interviewed using semi-structured questionnaires that focused on the treatment strategies, and the use of insecticides in the farms. Further, qualitative data was collected through direct observations, in-depth interviews and focus group discussions.

### Mosquito collections

Mosquito larvae were collected during the rainy season as well as before and during the period of insecticide treatments. The treatment periods started from July to October. During pest control, insecticide residues contaminate mosquito breeding sites whereby they diffuse into the water applying a selection pressure on mosquito larvae. Larvae were collected in the breeding sites of each site and transported to the laboratory of the Centre de Recherche Entomologique de Cotonou, Benin (CREC) for resistance testing. The adults were tested after emergence. A laboratory susceptible strain of *An. gambiae *Kisumu was used as a reference strain to compare the susceptibility levels of the field populations.

### Insecticide susceptibility tests

Mosquitoes collected were assayed using WHO discriminating dosages of four insecticides: permethrin (0.75%), DDT (4%), deltamethrin (0.05%) and bendiocarb (0.1%). Four batches of 25 unfed females, aged 2-5 days, were exposed to the diagnostic doses of insecticide treated papers for 1 hour. The twenty five females of *An. gambiae *were introduced into each tube and monitored at different time intervals (10, 15, 20, 30, 45, 60 minutes) the number "knocked-down" recorded. After one hour exposure, mosquitoes were transferred into holding tubes and provided with cotton wools wet with a 10% honey solution. Mortalities were recorded after 24 hours and the susceptibility status of the population was graded according to the WHO protocol [28]. Dead and surviving mosquitoes from this bioassay were separately kept in Carnoy solution at -20°C for further molecular analysis.

### Molecular characterization

All *An. gambiae *s.l. were identified to species using PCR [29] and as M and S forms by PCR-RFLP [30]. To detect the presence of *Kdr *mutation in the samples collected from each study site, polymerase chain reaction diagnostic test for detection of *kdr *"Leu-phe" genes was carried out on *An. gambiae *mosquitoes from each study site as described by Martinez-Torres et *al*. [31].The PCR-RFLP diagnostic test was used to detect the presence of G119S gene (*Ace.1 *gene) as described by Weill et *al*. [32].

### Screening of pesticide residues in soil from agricultural settings

This investigation was performed using indirect bioassays focused on factors which can affect the normal growth of mosquito larvae in cotton breeding sites. The hatching rates of *An. gambiae *eggs and the larval survival during rearing period were assessed in artificial breeding sites made of soil samples collected from different cotton areas. The pyrethroid-susceptible Kisumu strain and the resistant VKPER strain were used to test the presence of insecticide residues in the soil samples by means of mortality rates after exposure. The testing method was based on an artificial breeding site made of a mixture of soil from cotton sites (BP, TICP and CCP programs) and CREC soil used as a control. 100 g of each soil was weight and mixed in 1,000 ml of water. 200 eggs of the susceptible *Kisumu *strain were placed in each artificial breeding site and compared with 200 eggs of VKPER.

Larvae in all artificial breeding sites were fed with similar quantity and type of food (well ground cat biscuits mixed with yeast powder). Daily observation was done in order to record the number and the instars of larvae. This experiment was replicated three times per month.

### Data interpretation

The resistant status of mosquito samples was determined according to the WHO criteria [33]:

• Mortality rates is > 97%: the population was considered fully susceptible

• Mortality rates ranged between 80 > × < 97%: resistance suspected in the population

• Mortality rates < 80%, the population was considered resistant to the tested insecticides.

The knockdown times for 50% and 95% of tested mosquitoes (*KdT50 *and *KdT95*) were estimated using a log-time probit model [34].

The resistance allele frequency at the *kdr *and *Ace-*1 locus was calculated using Genepop software (version 3.3) as described by Raymond and Rousset [35].

A Fisher's exact test was performed to compare the resistance allele frequency at the *kdr *and *Ace-*1 among the mosquitoes from the different strategies.

An analysis of variance (ANOVA) was performed to compare the percentage of hatching eggs in the different treatments in order to know the impact of insecticide on the normal growth of mosquito larvae in cotton breeding sites.

## Results

### Knowledge-Attitude -Practice (KAP) investigations

Results from our KAP investigations from June to September 2008 in the cotton growing areas showed a common use by farmers of mineral fertilizer NPK at about 200 kg.ha-1 and urea at about 50 kg.ha-1 in both the CCP and the TICP sites. By contrast, in BP areas, all farmers in this group used organic and natural fertilizers which included animal excreta. In the CCP sites, about 6 pesticide treatments were applied by farmers 45 days after seeding and at two week intervals from flowering. Endosulfan or Tihan^® ^(mixture of spirotetramat + flubendiamide), were sprayed in the first two treatments followed by the mixtures of cyfluthrin + chlorpyrifos ethyl for the 3^rd ^and the 4^th ^treatment and then cypermethrin + dimethoate applied for the last two treatments. In TICP sites, the same treatments at intervals of two weeks were made either at half dose or at a complete full dosage when the threshold of infestation was reached (5 of *H. armigera *larvae observed on 50 plants). In areas practicing Biological Program, farmers apply a mixture of neem or papaya leaves with added chilli and local soap three times before the harvest.

### Resistance to insecticides

A total of 1,313 females of *An. gambiae *collected from different sites around Parakou, N'dali, Kandi, and Banikoara were exposed to papers impregnated with discriminating doses of permethrin (0.75%), deltamethrin (0.05%), DDT (4%) and bendiocarb (0.1%).

**Figure 2:**
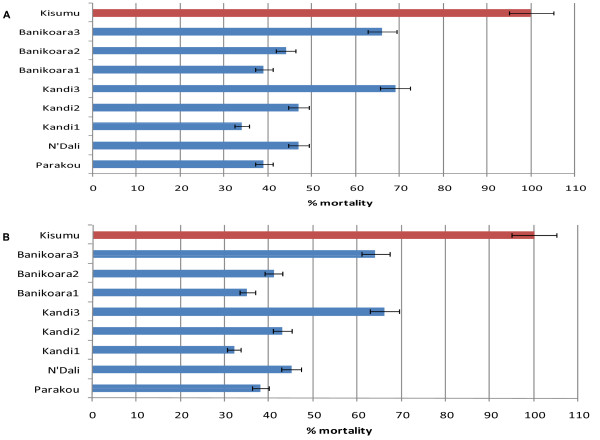
**Mortality rates and resistance status of *Anopheles gambiae s.l. *collected before (A) and after (B) cotton treatments to DDT 4% using WHO bioassay tests**.

**Figure 3:**
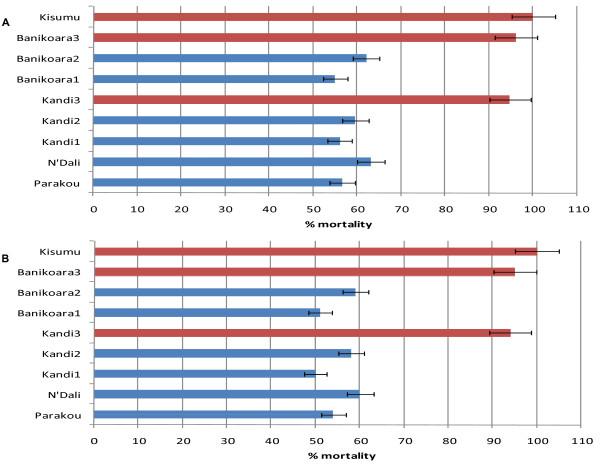
**Mortality rates and resistance status of *Anopheles gambiae s.l. *collected before(A) and after (B) cotton treatments to permethrin 0.75% using WHO bioassay tests**.

The knockdown times (*KdT50*, *KdT95*) of *An. gambiae *populations from CCP and TICP sites were significantly longer than that of the susceptible strain Kisumu (p < 0.05). However, the *KdT50 *for *An. gambiae *from BP site around Kandi was not significantly different from Kisumu (Table 1). Data recorded before and during the period of treatments showed a higher resistance to DDT and permethrin in populations from the CCP and TICP sites compared with those from the BP sites (Figure 2 and Figure 3). All populations of *An. gambiae *mosquitoes were resistant to DDT with an average of 33%, 42% and 65% of mortality respectively for CCP, TICP and BP sites. The mortality difference associated with the different pesticide application strategies was highly significant between BP and CCP programs (*P *< 0.05) but not significant between BP and TICP program (P = 0.56). However, *An. gambiae *populations from BP, CCP and TICP sites were fully susceptible to deltamethrin and bendiocarb (100% of mortality). Permethrin resistance was found in *An. gambiae *populations from CCP and TICP sites with an average mortality of 50% and 58% respectively. However, *An. gambiae *collected from BP sites were more susceptible to permethrin with 94% mortality.

**Table 1:** Knockdown times (*KdT*_50 _and *KdT*_95_) and mortality of *Anopheles gambiae *s.l. populations from 3 cotton sites after exposure to DDT 4% and permethrin 0.75% and their resistance status

Sites/Strains	Program	Insecticides	N	kdT50 [Cl95] (min)	kdT95 [Cl95] (min)	% Mortality [Conf lim 95]	Resistance status
		DDT	40	65.1 [57.5-73.4]	152.1 [118.4-228.1]	38 [29.31-46.7]	R
Parakou	CCP	Permethrin	75	35.3 [32.1-38.4]	112.1 [88.2-151.9]	54 [47.49-60.5]	R

		DDT	60	38.1 [29.4-36.5]	65.1 [57.5-86.5]	45 [37.73-52.3]	R
N'dali	TICP	Permethrin	60	19.3 [15.6-22.4]	67.1 [53.2-87.4]	60 [52.8-67.16]	R

		DDT	70	63.1 [60.2-72.3]	135.1 [5.2-184.5]	32 [25.7-38.31]	R
Kandi1	CCP	Permethrin	80	19.3 [15.6-22.4]	87.1 [63.5-138.4]	50 [43.67-56.32]	R

		DDT	50	35.1 [27.1-35.2]	62.5 [54.2-79.1]	43 [35.07-50.92]	R
Kandi 2	TICP	Permethrin	88	15.1 [13.6-20.1]	56.5 [43.2-77.2]	58 [52.00-64]	R

		DDT	60	30.3 [25.4-38.9]	72.6 [50.2-90.5]	66 [59.08-72.92]	R
Kandi 3	BP	Permethrin	80	11.0 [8.7 - 17.6]	23.5 [18.4-35.8]	94 [91.01-96.98]	S

		DDT	65	56.5 [51.7-63.2]	186.9 [146.0-265.2]	35 [28.3041.69]	R
Banikoara 1	CCP	Permethrin	90	24.6 [20.3-29.0]	105.2 [92-130.8]	51[45.04-56.96]	R

		DDT	70	32.4 [25.8-30.2]	60.1 [50.1-78.6]	41 [34.34-47.65]	R
Banikoara 2	TICP	Permethrin	70	18.5 [16.1-22.4]	58.1 [47.4-78.4]	59 [52.35-65.65]	R

		DDT	75	29.1 [24.4-37.6]	70.1 [48.2-88.2]	64 [57.73-70.27]	R
Banikoara 3	BP	Permethrin	80	13.2 [9.4 - 18.2]	25.5 [20.2-37.1]	95 [92.24-97.75]	S

		DDT	100	25.7 [24.3-27.0]	40.7 [38.0-44.5]	98 [96.41-99.58]	S
*An. gambiae *s.l	*	Permethrin	100	10.9 [9.7-12.0]	18.1[16.0-21.6]	99 [97.87100.13]	S

*KdT*_*50 *_, knockdown time in min for 50% mosquitoes; *KdT*95 , knockdown time in min for 95% mosquitoes; CI, confidence interval at 95%;

*, No program = Control; Conf Lim 95% = confidence interval at 95%.

### Species identification

A total of 850 *An. gambiae *adults were analysed for species and molecular forms. Most of the mosquitoes collected from all the sites were *An. gambiae *s.s. (90%), which was found in sympatric with a low proportion of *An. arabiensis *(0 to 5%) except in Parakou and N'dali where they were more present (22 to 30% respectively) (Table 2). In *An. gambiae s.s*, the M and S forms were always found in sympatric but the S form was mostly predominant (96%).

**Table 2:** Species and molecular forms identification within *Anopheles gambiae *complex and the frequency of *Kdr *and *Ace-1R *mutations in *Anopheles gambiae *s.s. in Benin

	**Species**^**a**^		Mol. Form		*Kdr *mutation				*Ace.1 *mutation			
**Locality**	**%Aa**	**%Ag**	**%M**	**%S**	**SS**	**RS**	**RR**	**F(R)**	**SS**	**RS**	**RR**	**F(R)**

Parakou^1^(92)	22	78	5	95	12	35	45	0.68	30	02	0	0.06
N'dali^2^(92)	30	70	3	97	22	40	30	0.54	25	0	0	0.00
Kandi^1^(92)	5	95	0	100	7	45	40	0.67	30	02	0	0,06
Kandi^2^(92)	4	96	0	100	28	34	30	0.51	30	01	0	0.03
Kandi^3^(90)	0	100	0	100	45	25	20	0.35	30	0	0	0.00
Banikoara^1^(102)	3	97	0	100	12	36	54	0.78	30	02	0	0.06
Banikoara^2^(92)	02	98	0	100	14	48	30	0.59	30	0	0	0.00
Banikoara^3^(96)	02	98	2	98	48	36	12	0.32	25	0	0	0.00

( a) Aa = *An. arabiensis*; Ag = *An. gambiae s.s*., superscripts in column; 1 = Control strategies: 1 = CCP; 2 = TICP; 3 = BP

### Resistance mutations

The *kdr *genotype was scored for 1,400 individuals (100 mosquitoes consistently failed to amplify). The *kdr *gene occurred in S forms (Table 2). The highest frequency of *Kdr *mutation was recorded for the populations from three CCP sites (67-78%) and the lowest (35 and 32%) were found in the populations from BP sites around Kandi and Banikora respectively.

The *Ace-1R *gene was found at very low frequency ranging (from 0.00 to 0.06) in heterozygote *An. gambiae *s.s from the three CCP sites (Table 2). Among *An. gambiae *s.s, there was no mosquito of the M molecular form carrying the *ace-1R *gene.

The resistance allele frequency at the *kdr *was significantly higher in areas where farmers used insecticide for pest control (CCP and TICP) than in those no insecticide is not request (BP) (p < 0.05.). However, there is no difference between the resistance allele frequency at the *kdr *from mosquitoes in CCP and TICP strategies (p > 0.05).

### Pesticide residues

**Figure 4:**
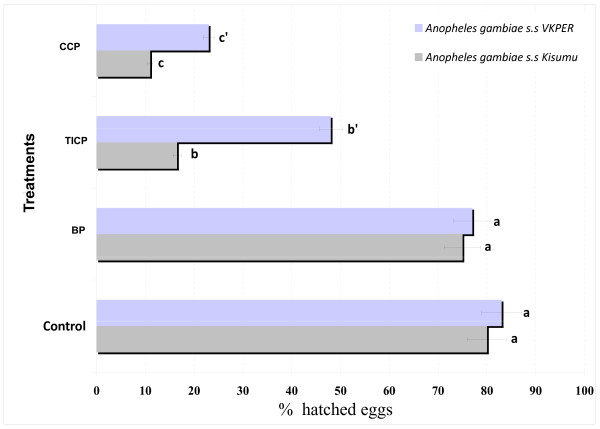
**Percentage of eggs hatching of *An. gambiae *in water collected from breeding sites inside CCP program (in Parakou), TICP program (in N'Dali), BP program (in Banikoara) compared with distilled water (as control)**.

Results of soil samples for pesticide residues analysis showed that artificial breeding sites made with soil from CREC (control) and soil from Biological program (BP) sites were similar with no effect on hatching of *An. gambiae *Kisumu and VKPER strains (Figure 4). Tests with the susceptible *An. gambiae *Kisumu strain gave percentages of hatching equivalent to 80% in control soil (no contact with pesticides) and 75% with soil from BP sites. However, with the pyrethroid resistant strain VKPER the percentages of hatching were 83% and 77% with the control soil and soil from BP sites respectively. The hatching percentages of both strains decreased significantly when soil samples from CCP and TICP sites were used. With the TICP soil, *VKPER *hatching was 45% against 25% for the Kisumu strain, whiles the CCP soil gave 34% hatching for VKPER and 11% for Kisumu. In both cases the results showed that the hatching rates were significantly higher (P < 0.05) with VKPER than Kisumu when the soil samples tested were from TICP and CCP sites.

**Figure 5:**
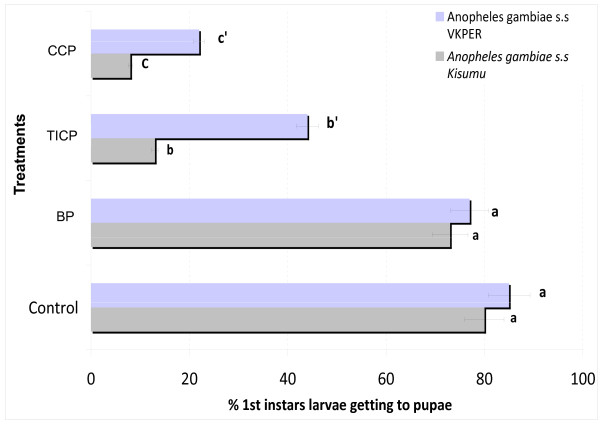
**Percentage of emerging adults of *An gambiae *reared in water collected from breeding sites inside CCP program (in Parakou), TICP program (in N'Dali), BP program (in Banikoara) compared with distilled water (as control)**. - **A**: before treatments - **B**: after treatments • Areas for CCP programs: Parakou; Kandi 1 and Banikoara1 • Areas for TICP programs: N'dali, Kandi 2 and Banikoara 2 • Areas for BP programs: Kandi 3 and Banikoara 3

Similar results were obtained with the emergence of adults of *VKPER *and Kisumu strains from eggs placed in artificial breeding sites consisting of water and soil samples from CCP and TICP relative to the control (Figure 4). There was no significant difference between the emergence of adults of *VKPER *and *Kisumu *strains breeding in artificial sites made with the soil samples from BP compared with the control (Figure 5).

However, a significant difference (P < 0.05) was observed between the emergence of *VKPER *on artificial sites made of soils from TICP (43%) and CCP (35%) sites and Kisumu which gave 20% emergence on TICP and 13% on CCP.

## Discussion

The information generated during interviews with cotton farmers and the observations made in cotton fields has confirmed a common use of fertilizers and insecticides in cotton fields. Cotton cultivation requires intensive use of pesticides including insecticides belonging to the two main classes recommended for vector control in public health: organophosphates and pyrethroids. In West Africa, pyrethroid-treated bed nets remain one of the effective tools for malaria vector control and it provides personal protection to individuals who sleep under them. When used by the whole community, bed nets protect collectively against infective mosquito bites by a mass killing effect of the vectors [36].

In Benin, pyrethroids have been extensively introduced in agriculture since 1980s [25]. This factor is probably one of the causes of the selection of strong resistance in *An. gambiae *to permethrin and DDT, particularly in cotton growing areas. Based on recent results, several authors [8-12; 37] have reported that past and current agricultural use of DDT then pyrethroids for crop protection have led to the selection of resistant mosquitoes through insecticide residues accumulated in breeding sites around cotton growing areas. This hypothesis was recently confirmed by Akogbeto *et al. *[25] showing indirectly the presence of pesticide residues in soil and water from vegetable farms and other agricultural activities in Benin that delay or reduce the emergence rates of mosquito larvae.

The use of insecticides in households for public health purposes and massive quantities of pesticides in agricultural settings has been highlighted as a key factor contributing to the emergence of vector resistance. A recent report by Yadouleton et *al*.[37] showed that agricultural practices in urban areas seem to have contributed to the emergence of insecticide resistance in *Anopheles *populations. Our study in vegetable farming systems in Benin demonstrated that improper use of insecticides to control vegetable pests in urban areas directly exerted a huge selection pressure on mosquito larval populations. The high mortality observed with mosquitoes reared on soils from CCP, TICP sites can be explained by the presence of DDT residues in the soil from those sites and the extensive use of pyrethroids by farmers [38]. Our results showed that the high level of the *kdr-west (*Leu-Phe) gene seemed to be the main resistance mechanism and responsible for the decrease of mortality rates to DDT and permethrin and is more of an ongoing process in *An. gambiae *populations from CCP and TICP sites. The *kdr *gene in the main malaria vector *An. gambiae *was found at high frequency in samples from the sites using insecticide (CCP and TICP) than those with no use of insecticide (BP program). The low frequency of *Kdr *gene in BP localities compared with those from CCP and TICP could be due to the fact that in the past these farmers in BP sites used insecticide to control cotton pests. According to reports by Akogbeto et *al *[25], Djogbénou et *al *[14], and Yadouleton et *al. *[38], the presence of *Kdr *genes in mosquito can be due to external factors that affect mosquitoes as larvae or adults. In 2000, a study in Burkina Faso by Diabate *et al. *[17] reported higher levels of *kdr *alleles frequency in *An. gambiae *collected from cotton-growing areas constantly subjected to insecticide treatments, as compared to the low frequency of *kdr *recorded in rural areas where farmers are restricted to low or no use of pesticides. Despite the use of insecticide in both CCP and TICP sites, the difference in adult mortality rates between CCP and TICP program can be explained by the fact that CCP program uses more insecticide than TICP program.

This study provides clear evidence of the association between the use of insecticides in agriculture and the widespread emergence of insecticide resistance in *Anopheles *species.

Indeed, in Benin, insecticide treatments against pests in cotton plantations are carried out twice a month, for an average period time of three months (between July and October) per year. That treatment period during the rainy season correspond with the period of high mosquito densities because *Anopheles *populations have numerous breeding sites particularly in cultivated areas. As reported by Akogbeto *et al *[26], some populations of *An. gambiae *may lay their eggs in breeding sites containing insecticide residues. These eggs undergo a selection pressure from agricultural pesticides, which leads to the emergence of resistant strains. There is clear evidence on the implication of agricultural use of insecticides in the selection of resistance in the major malaria vectors. Our results agree with the work of Akogbeto *et al *[25] and confirm once again the impact of the extensive use of insecticides in cotton crop protection on the emergence of insecticide resistance in *An. gambiae *populations. Moreover, in CCP and TICP program, some farmers used insecticides belonging to the organophosphate classes. *Ace-1R *gene is the main resistance mechanism of *An. gambiae *s.l. to organophosphates and carbamates also. The present study, has shown that the *ace-1R *gene is present at low frequency (ranging from 0.01 to 0.09), but only in CCP and TICP program. However, previous field surveys on *An. gambiae s.l. *populations of South-Western

Burkina Faso by Djogbenou *et al *[14] in cotton fields showed that *ace-1R *gene was the main resistance mechanism in *An. gambiae *s.l.

However the National Malaria Control Program (NMCP) in Benin has started scaling up Long Lasting Insecticidal Nets (LLINs) and carbamate for Indoor Residual Spraying (IRS) countrywide for malaria control. The challenge to find effective strategies to manage insecticide resistance in *Anopheles gambiae *remains a high priority and an urgent need particularly in Benin where pyrethroid resistance has been reported with a clear evidence in experimental huts of reduced efficacy of ITNs and IRS [39]. One of the strategies will be to remove pyrethroids from agricultural pest control and leave these classes of insecticides for public health purposes and promote other classes of insecticides such as Spinosad which does not show cross resistance to pyrethroids (i.e. the *kdr *gene).

## Conclusions

With the spread of *Kdr *allele frequency from CCP and TICP programmes, to reduce the emergence of insecticide resistance in *An. gambiae *population, African governments would be better advised to promote the BP cotton or genetically modified cotton such as *Bt *Cotton (*Bacillus thurengiensis*) which require lower pesticide than the cotton with CCP and TICP programme and would permit to suppress the massive use of pyrethroid insecticides.

## Competing interests

The authors declare that they have no competing interests.

## Authors' contributions

AY carried out field experiments, collected, analysed, interpreted data and wrote the manuscript. TM, GP reviewed the manuscript and contributed to the design of the study and substantially helped in drafting the manuscript, and revised the manuscript. FC**, **LD, RD, IG and MB contributed to the design of the study. AA helped in drafting and reviewing the manuscript and RA helped with the activities. TM, MA conceived and designed the study, supervised fields and laboratory procedures, and reads the manuscripts. All authors read and approved the final manuscript.
